# Draft Genome Sequence of a New *Bacillus cereus* Strain tsu1

**DOI:** 10.1128/genomeA.01294-14

**Published:** 2014-12-18

**Authors:** Hui Li, Suping Zhou, Terrance Johnson, Koen Vercruysse, Alexander J. Ropelewski, Theodore W. Thannhauser

**Affiliations:** a College of Agriculture, Human and Natural Sciences, Tennessee State University, Nashville, Tennessee, USA; b Pittsburgh Supercomputer Center, Pittsburgh, Pennsylvania, USA; c R.W. Holley Center for Agriculture and Health, Plant, Soil and Nutrition Research Unit, USDA, ARS, Ithaca, New York, USA

## Abstract

This paper reports the draft genome sequence of a new *Bacillus cereus* strain tsu1, isolated on an agar-cellulose plate. The draft genome sequence is 5.81 Mb, revealing 5,673 coding sequences. It contains genes for cellulose-degradation and biosynthesis pathways of polyhydroxybutyrate (PHB) and 8 rRNA genes (5S, 16S, and 23S).

## GENOME ANNOUNCEMENT

*Bacillus cereus* is a widespread bacterium dwelling in soil. Several strains have demonstrated biochemical activities in degrading cellulose and also producing valuable biopolymers. Whole-genome sequencing of these strains will provide significant resources for developing sustainable bioenergy and reducing the reliance on petroleum-based plastics.

In this study, we present the genome sequence and annotation of a new *B. cereus* strain tsu1. The strain, designated *B. cereus* strain tsu1, was isolated from a cellulose-agar plate. The bacterium is a Gram-positive, rod-shaped organism with a centrally located endospore, which is identical to the *B. cereus* species. Extracellular cellulase activity was observed when the bacteria were cultivated on 10% carboxymethyl cellulose (Acros Organics, NJ) agar plates followed by Congo-red staining (1). The extracellular cellulase proteins were identified using mass-spectrometry analysis of the proteins secreted into the growth broth containing cellulose substrate. Proteins were isolated by precipitation with 10% trichloroacetic acid in acetone followed by separation by sodium dodecyl sulfate polyacrylamide gel electrophoresis. Coomassie blue stained protein bands were excised and digested with trypsin and analyzed by liquid chromatography (LC)/mass spectrometry (MS) (2). Mascot v2.3 was used to search the mass spectrometry data against NCBInr (firmicutes restriction). The PHB producing activity was confirmed using Sudan-black staining (3) and ion-exclusion high-pressure liquid chromatography analysis (4).

Genomic DNA was extracted from the abovementioned strain using the GenElute Bacterial genomic DNA kits for Gram-positive strains (Sigma, CA) according to the manufacturer’s instructions. The genome was sequenced on an Illumina HiSeq 2000 using TruSeq SBS kit v3 for paired-end 100 bp sequencing at the Genome Facility at Cornell University. For data quality control, the length of each read was trimmed with FASTX from the 3′ end of the read using a quality threshold of 20. The draft genome sequence was assembled into 67 contigs using velvet, Kmer = 79 (version 1.2.10), and then into 36 scaffolds using SSPACE (5) followed by CAP3 (6).

The draft genome sequence of *Bacillus cereus* strain tsu1 is 5.81 Mb in size with a G+C content of 34.8%. The genome contains a PhaA gene (beta keto-thiolase), PadR, PhaB, PhaC_N, and PHB_depo_C, and cellulase and hemicellulase genes. The 16s rRNA gene has a 99% identity to *B. cereus* and *B. thuringiensis*.

### Nucleotide sequence accession number.

This whole-genome project has been deposited in NCBI/GenBank under the accession no. JPYN00000000. The version described in this paper is the first version.

## References

[1] Jakucs E Várallyay E 1995 A simple and rapid semiquantitative method for measuring cellulase activity in agar media. Acta Microbiol. Immunol. Hung. 42:77–80. 7542535

[2] Yang Y Zhang A Howe K Wilson DB Moser F Irwin D Thannhauser TW 2007 A comparison of nLC-ESI-MS/MS and nLC-MALDI MS/MS for GeLC-based protein identification and iTRAQ-based shotgun quantitative proteomics. J. Biomol. Tech. 18:226–237. 17916795 PMC2062563

[3] Wei YH Chen WC Huang CK Wu HS Sun YM Lo CW Janarthanan OM 2011 Screening and evaluation of polyhydroxybutyrate-producing strains from indigenous isolate *Cupriavidus taiwanensis* strains. Int. J. Mol. Sci. 12:252–265. 10.3390/ijms12010252. 21339985 PMC3039951

[4] Karr DB Waters JK Emerich DW 1983 Analysis of poly-beta-hydroxybutyrate in *Rhizobium japonicum* bacteroids by ion-exclusion high-pressure liquid chromatography and UV detection. Appl. Environ. Microbiol. 46:1339–1344. 16346443 10.1128/aem.46.6.1339-1344.1983 PMC239573

[5] Boetzer M Henkel CV Jansen HJ Butler D Pirovano W 2011 Scaffolding pre-assembled contigs using SSPACE. Bioinformatics 27:578–579. 10.1093/bioinformatics/btq683. 21149342

[6] Huang X Madan A 1999 CAP3: a DNA sequence assembly program. Genome Res. 9:868–877. 10.1101/gr.9.9.868. 10508846 PMC310812

